# Supplementary material for: “When in Doubt, Change It out”: A Case-Based Simulation for Pediatric Residents Caring for Hospitalized Tracheostomy-Dependent Children
Source: MedEdPORTAL. 2020 Oct 1;16:10994. doi: 10.15766/mep_2374-8265.10994 (PMC7528672; doi:10.15766/mep_2374-8265.10994)
Supplement: Supplementary file 1 — Simulation Case 1 Template.docxSimulation Case 2 Template.docxSimulation Case 3 Template.docxAssessment Score Sheet.docxCase Scenario Visual Cards.docxSimulation Feedback Tool.docx [file mep_2374-8265.10994-s001.zip › A. Simulation Case 1 Template.docx]

| Appendix A: Simulation Case #1 Template  SIMULATION CASE TITLE: Scenario #1: Tracheostomy and ventilator-dependent patient with vent malfunction, trach complications  AUTHORS: Khan EK, MD; Lockspeiser TM, MD; Liptzin DR, MD, MS; Baker CD, MD  **LEARNER AUDIENCE:** Pediatric Resident Physicians | |
| --- | --- |
| **PATIENT NAME:** Baby Ruth  **PATIENT AGE:** 10 months  **CHIEF COMPLAINT:** Low-pressure ventilator alarm  **PHYSICAL SETTING:** In classroom, empty patient room, or simulation center. Mannequin with tracheostomy in place, ventilator disconnected with “low pressure” alarm simulation, suction available (shown here with in-line suction in place).  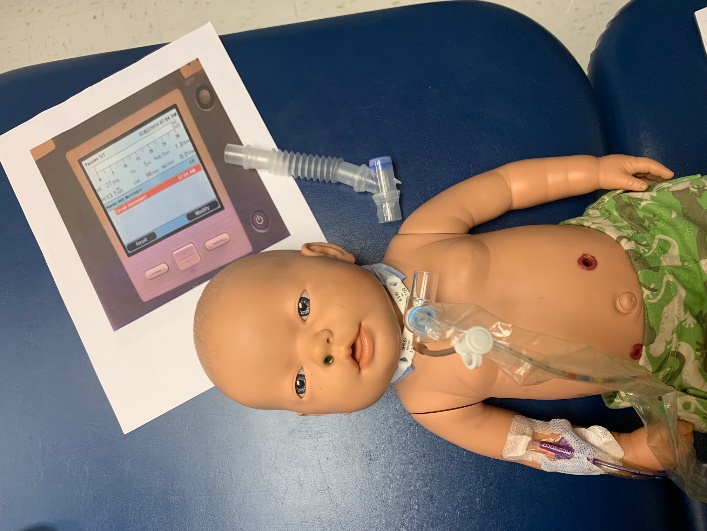  *Image Citation: Author Owned* | |
| **Brief narrative description of case** | This scenario involves a tracheostomy and ventilator dependent patient who experiences ventilator malfunction in the form of ventilator disconnect, followed by tracheostomy complications of tube obstruction requiring suctioning and changing the tracheostomy tube. The final required intervention in this scenario is to increase oxygen in response to hypoxemia. |
| **Primary Learning Objectives** | 1. Identify when the child’s status is deteriorating based on color change, neurological response, work of breathing, and ventilator alarm status. 2. Demonstrate troubleshooting in setting of high- and low-pressure ventilator alarms. 3. Demonstrate correct steps to alleviate a plugged trach tube by suctioning and changing a tracheostomy tube and increasing oxygen flow. |
| **Critical Actions** | Stress importance of checking stoma for tracheostomy tube placement, equipment connections, suctioning, and “when in doubt, change it out” |
| **Learner Preparation or Prework** | None |

| Initial Presentation: “You just finished rounding and are heading to do some physical exams. You walk into your favorite patient’s room and hear the ventilator alarm.” | | | |
| --- | --- | --- | --- |
| **Initial vital signs** | See Appendix D Case Scenario Visual Cards: vital signs 1.1  HR 170/min  BP 90/50  RR 45/min  Sat 89% on RA | | |
| **Overall Setting and Appearance** | See Appendix D Case Scenario Visual Cards: vital signs 1.1  Dusky  Anxious  Increased WOB  Slightly diminished aeration with crackles and rhonchi | | |
| **Confederates (e.g., standardized participants) and their roles in the room at case start** | Facilitator: to guide learner through case stages  Learner(s): 1-3 resident physicians, each taking turns “leading” each scenario while others provided back-up as “helpers” when called upon by lead resident | | |
| **HPI** | This is a 10-month-old former 26-week premature infant with tracheostomy and ventilator dependence. She has been in usual state of health, awaiting discharge criteria of educating home providers. | | |
| **Past Medical/Surgical History** | **Medications** | **Allergies** | **Family History** |
| 26-week prematurity with uncomplicated NICU course, mild well controlled pulmonary hypertension, mild reflux, gastrostomy tube dependence | Sildenafil | None | None |
| Physical Examination - See Appendix D Case Scenario Visual Cards (intentionally limited) | | | |

| Instructor Notes - Changes and CASE Branch Points | | | | |  |  |
| --- | --- | --- | --- | --- | --- | --- |
| STAGE | | VITAL SIGNS 1.1 | PHYSICAL EXAM | PARTICIPANTS’ REQUIRED ACTS | NOTES TO OPERATOR | |
| Initial assessment | | HR 170/min  BP 90/50  RR 45/min  Sat 89% on RA | Dusky  Anxious  Increased WOB  Slightly diminished aeration with crackles and rhonchi | Check airway/trach/ventilator connections.   1. Does not perform correctly 2. Performs correctly with guidance or prompting 3. Performs correctly without assistance *[visualizes trach entering stoma, checks ventilator connection]* | Home ventilator, simulate low pressuring  Tracheostomy in place, ventilator disconnected  Suction available | |
| STAGE | | VITAL SIGNS 1.1 | PHYSICAL EXAM | PARTICIPANTS’ REQUIRED ACTS | NOTES TO OPERATOR | |
| Intervention: Reconnect ventilator | | HR 170/min  BP 90/50  RR 45/min  Sat 89% on RA | Dusky  Anxious  Increased WOB  Slightly diminished aeration with crackles and rhonchi | Replaces ventilator connection.   1. Does not perform correctly 2. Performs correctly with guidance or prompting 3. Performs correctly without assistance *[establishes ventilator connection]* | Home ventilator, simulate low pressuring  If resident moves to any other step, prompt: What do you think about the ventilator alarm? And/or Would you like to check all the connections before moving on? | |
| STAGE | | VITAL SIGNS 1.2 | PHYSICAL EXAM | PARTICIPANTS’ REQUIRED ACTS | NOTES TO OPERATOR | |
| Intervention: Suctioning | | HR 175/min  BP 80/40  RR 60/min  Sat 82% on RA | Blue  Anxious  Still with diminished aeration | Responds to high vent alarm by suctioning.   1. Does not perform correctly 2. Performs correctly with guidance or prompting 3. Performs correctly without assistance *[suctions with sterile technique]* | Home ventilator, simulate high pressuring  Resident suctions trach and catheter passes freely, mucus plug removed from trach  If resident moves to any other step, prompt: Would you like to try suctioning before moving on? | |
| STAGE | | VITAL SIGNS 1.2 | PHYSICAL EXAM | PARTICIPANTS’ REQUIRED ACTS | NOTES TO OPERATOR | |
| Intervention: Change trach | | HR 175/min  BP 80/40  RR 60/min  Sat 82% on RA | Blue  Anxious  Still with diminished aeration | Responds to patient by changing trach.   1. Does not perform correctly 2. Performs correctly with guidance or prompting 3. Performs correctly without assistance *[changes trach with assistance from “helper”]* | Resident notes no improvement in patient or vital signs, moves to change trach tube.  If resident moves to any other step or unsure of what to do, prompt: Do you believe the airway is fully intact? And/or Would you like to try changing the tracheostomy tube? | |
| STAGE | | VITAL SIGNS 1.3 | PHYSICAL EXAM | PARTICIPANTS’ REQUIRED ACTS | NOTES TO OPERATOR | |
| Intervention: Increasing oxygen | | HR 120/min  BP 90/50  RR 40/min  Sat 82% on RA | Awake, alert  Breathing comfortably with good aeration and good chest rise, symmetrical exam | Responds to hypoxemia.   1. Does not perform correctly 2. Performs correctly with guidance or prompting 3. Performs correctly without assistance *[adds supplemental O2]* | Resident to start supplemental oxygen, saturations increase.  If resident moves to any other step or unsure of what to do, prompt: What is your impression of the patient’s oxygen saturation? And/or Would you add supplemental oxygen? | |
| STAGE | | VITAL SIGNS 1.4 | PHYSICAL EXAM | PARTICIPANTS’ REQUIRED ACTS | NOTES TO OPERATOR | |
| Child recovers | | HR 110/min  BP 90/50  RR 30/min  Sat 95% on 2LPM bled in | Happy and playful  Back to baseline |  | End of simulation, enter debriefing. | |

Debriefing:

1. Description: Reinforce safe learning environment, focus on what happened

Example questions: So, what happened? How did that go?

1. Analysis: Analyze and explore what happened in detail

Example questions: What do you think was going on when the patient had high pressure alarms?

1. Application: Move from specifics of this case to general practice

Example questions: What else could have been going on? What would you have done if the patient didn’t respond to the tracheostomy change?

**Ideal Scenario Flow:**

The learner enters patient room and provides initial assessment - visualizes trach entering stoma, checks ventilator connection, notes low-pressure ventilator alarm notification. The learner recognizes disconnected ventilator tubing and replaces the connections. The learner then notes the high-pressure ventilator alarm notification, recognizes tracheostomy tube obstruction, and suctions with sterile technique. Patient does not improve with suctioning, therefore learner changes tracheostomy tube with assistance from “helper”. Patient improves and begins to recover but remains hypoxemic. The learner recognizes hypoxemia and recommends supplemental oxygen therapy.

**Anticipated Management Mistakes: (see above “Notes to Operator”)**

1. Failure to visualize tracheostomy tube entering stoma: Many learners will not visualize the tube entering the stoma and often needed to be reminded of this key step.
2. Delay in initiating oxygen therapy: Occasionally learners would be slow to oxygen therapy. We addressed this during the session with prompting questions as listed above.
