# Supplementary material for: “When in Doubt, Change It out”: A Case-Based Simulation for Pediatric Residents Caring for Hospitalized Tracheostomy-Dependent Children
Source: MedEdPORTAL. 2020 Oct 1;16:10994. doi: 10.15766/mep_2374-8265.10994 (PMC7528672; doi:10.15766/mep_2374-8265.10994)
Supplement: Supplementary file 1 — Simulation Case 1 Template.docxSimulation Case 2 Template.docxSimulation Case 3 Template.docxAssessment Score Sheet.docxCase Scenario Visual Cards.docxSimulation Feedback Tool.docx [file mep_2374-8265.10994-s001.zip › B. Simulation Case 2 Template.docx]

| Appendix B: Simulation Case #2 Template  SIMULATION CASE TITLE: Scenario #2: Tracheostomy-dependent patient with accidental decannulation  AUTHORS: Khan EK, MD; Lockspeiser TM, MD; Liptzin DR, MD, MS; Baker CD, MD  **LEARNER AUDIENCE:** Pediatric Resident Physicians | |
| --- | --- |
| **PATIENT NAME:** Baby Ruth  **PATIENT AGE:** 10 months  **CHIEF COMPLAINT:** Increased work of breathing  **PHYSICAL SETTING:** In classroom, empty patient room, or simulation center. Mannequin with decannulated tracheostomy tube stuck under trach ties.  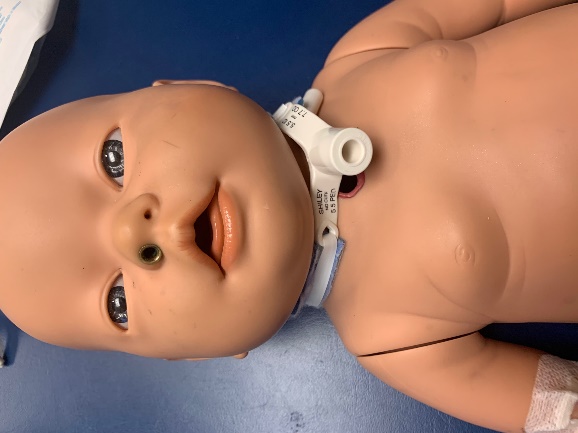  *Image Citation: Author Owned* | |
| **Brief narrative description of case** | This scenario involves a tracheostomy-dependent patient who experiences accidental decannulation of the tracheostomy tube requiring replacement of the tube, suctioning, and changing the tracheostomy tube. The final required intervention in this scenario is to provide bag-trach ventilation in response to poor respiratory effort. |
| **Primary Learning Objectives** | 1. Identify when the child’s status is deteriorating based on color change, neurological response, coughing, and work of breathing. 2. Demonstrate correct steps to reestablishing patent airway following decannulation. 3. Demonstrate emergency action steps including bag-trach ventilation. |
| **Critical Actions** | Stress importance of knowing when a patient has a critical upper airway, checking stoma for tracheostomy tube placement, suctioning, and “when in doubt, change it out” |
| **Learner Preparation or Prework** | None |

| Initial Presentation: “It’s 0300 during your call shift, and you are headed to evaluate a patient with asthma needing spacing of albuterol. While walking down the hallway, you encounter a nurse who stepped into the hallway to call for help. The nurse states she was doing routine vitals check and diaper change when she noticed that the patient looks pale, anxious, and is working harder to breathe.” | | | |
| --- | --- | --- | --- |
| **Initial vital signs** | See Appendix D Case Scenario Visual Cards: vital signs 2.1  HR 170/min  BP 100/60  RR 55/min  Sat 83% on 2LPM | | |
| **Overall Setting and Appearance** | See Appendix D Case Scenario Visual Cards: vital signs 2.1  Pale/Dusky  Anxious  Eyes open  Increased WOB  Extremely diminished aeration | | |
| **Confederates (e.g., standardized participants) and their roles in the room at case start** | Facilitator: to guide learner through case stages  Learner(s): 1-3 resident physicians, each taking turns “leading” each scenario while others provided back-up as “helpers” when called upon by lead resident | | |
| **HPI** | This is a 10-month-old term infant with subglottic stenosis secondary to traumatic intubation who is now tracheostomy-dependent. He has been in usual state of health, awaiting discharge criteria of educating home providers. | | |
| **Past Medical/Surgical History** | **Medications** | **Allergies** | **Family History** |
| Term, previously healthy, intubated 2 months ago in setting of RSV infection with respiratory failure, critical upper airway | None | None | None |
| Physical Examination - See Appendix D Case Scenario Visual Cards (intentionally limited) | | | |

| Instructor Notes - Changes and CASE Branch Points | | | | |  |  |
| --- | --- | --- | --- | --- | --- | --- |
| STAGE | | VITAL SIGNS 2.1 | PHYSICAL EXAM | PARTICIPANTS’ REQUIRED ACTS | NOTES TO OPERATOR | |
| Initial assessment | | HR 170/min  BP 100/60  RR 55/min  Sat 83% on 2LPM | Pale/Dusky  Anxious  Eyes open  Increased WOB  Extremely diminished aeration | Check airway/trach.   1. Does not perform correctly 2. Performs correctly with guidance or prompting 3. Performs correctly without assistance *[visualizes trach entering stoma]* | Child to have decannulated tracheostomy tube with tube stuck under trach ties. Suction available.  If resident moves to any other step, prompt: Do you believe the airway is fully intact? And/or Is the tracheostomy tube entering the stoma? | |
| STAGE | | VITAL SIGNS 2.2 | PHYSICAL EXAM | PARTICIPANTS’ REQUIRED ACTS | NOTES TO OPERATOR | |
| Intervention: Replace decannulated trach | | HR 175/min  BP 100/60  RR 60/min  Sat 80% on 2LPM | Blue  Anxious  Eyes open  Increased WOB  Extremely diminished aeration | Replaces trach.   1. Does not perform correctly 2. Performs correctly with guidance or prompting 3. Performs correctly without assistance *[replaces dislodged trach]* | Resident recognizes that child is decannulated and replaces trach. Resident moves to using obturator if unable to replace trach.  If resident moves to any other step, prompt: Would you like to try re-inserting the displaced tracheostomy tube first? | |
| STAGE | | VITAL SIGNS 2.2 | PHYSICAL EXAM | PARTICIPANTS’ REQUIRED ACTS | NOTES TO OPERATOR | |
| Intervention: Suctioning | | HR 175/min  BP 100/60  RR 60/min  Sat 80% on 2LPM | Blue  Anxious  Eyes open  Increased WOB  Extremely diminished aeration | Resident suctions trach.   1. Does not perform correctly 2. Performs correctly with guidance or prompting 3. Performs correctly without assistance *[suctions with sterile technique]* | Resident recognizes that child’s status is unchanged and suctions trach.  If resident moves to any other step, prompt: Would you like to try suctioning before moving on? | |
| STAGE | | VITAL SIGNS 2.2 | PHYSICAL EXAM | PARTICIPANTS’ REQUIRED ACTS | NOTES TO OPERATOR | |
| Intervention: Change trach | | HR 175/min  BP 100/60  RR 60/min  Sat 80% on 2LPM | Blue  Anxious  Eyes open  Increased WOB  Extremely diminished aeration | Changes trach.   1. Does not perform correctly 2. Performs correctly with guidance or prompting 3. Performs correctly without assistance *[changes to new trach with “helper”]* | Resident recognizes that child’s status is unchanged and changes trach.  If resident moves to any other step or unsure of what to do, prompt: Do you believe the airway is fully intact? And/or Would you like to try changing the tracheostomy tube? | |
| STAGE | | VITAL SIGNS 2.3 | PHYSICAL EXAM | PARTICIPANTS’ REQUIRED ACTS | NOTES TO OPERATOR | |
| Intervention: Bag-trach ventilation | | HR 160/min  BP 90/55  RR 50/min  Sat 80% on 2LPM | Blue  Anxious  Eyes open  Increased WOB  Somewhat improved aeration but still diminished | Bag-trach ventilation.   1. Does not perform correctly 2. Performs correctly with guidance or prompting 3. Performs correctly without assistance *[bags through trach, may ask for advice on PEEP settings]* | Resident begins bag-trach ventilation and child begins to recover.  If resident moves to any other step or unsure of what to do, prompt: Do you believe the patient is ventilating well? And/or Would you like to try giving a few breaths through the tracheostomy tube? | |
| STAGE | | VITAL SIGNS 2.4 | PHYSICAL EXAM | PARTICIPANTS’ REQUIRED ACTS | NOTES TO OPERATOR | |
| Child recovers | | HR 140/min  BP 100/60  RR 40  Sat 97% | Improved color  Improved aeration | Resident can stop bag-trach ventilation and put child back on trach collar set up. | End of simulation, enter debriefing. | |

Debriefing:

1. Description: Reinforce safe learning environment, focus on what happened

Example questions: So, what happened? How did that go?

1. Analysis: Analyze and explore what happened in detail

Example questions: Why do you think the patient needed bagging?

1. Application: Move from specifics of this case to general practice

Example questions: What else could have been going on? What would you have done if the patient didn’t respond to the bag-trach ventilation?

**Ideal Scenario Flow:**

The learner enters patient room and provides initial assessment - visualizes trach entering stoma. The learner recognizes tracheostomy tube decannulation and replaces the dislodged tube. The learner notes no improvement with replacement of tube and suctions with sterile technique. Patient does not improve with suctioning, therefore learner changes tracheostomy tube with assistance from “helper”. When the patient does not recover with tracheostomy change as noted by poor ventilation status, learner provides bag-trach ventilation. Patient improves and begins to recover; resident stops interventions.

**Anticipated Management Mistakes: (see above “Notes to Operator”)**

1. Failure to visualize tracheostomy tube entering stoma: Many learners will not visualize the tube entering the stoma and often needed to be reminded of this key step.
2. Delay in initiating bag-trach ventilation: Occasionally learners would be slow to initiate bag-trach ventilation. We addressed this during the session with prompting questions as listed above.
