# Supplementary material for: “When in Doubt, Change It out”: A Case-Based Simulation for Pediatric Residents Caring for Hospitalized Tracheostomy-Dependent Children
Source: MedEdPORTAL. 2020 Oct 1;16:10994. doi: 10.15766/mep_2374-8265.10994 (PMC7528672; doi:10.15766/mep_2374-8265.10994)
Supplement: Supplementary file 1 — Simulation Case 1 Template.docxSimulation Case 2 Template.docxSimulation Case 3 Template.docxAssessment Score Sheet.docxCase Scenario Visual Cards.docxSimulation Feedback Tool.docx [file mep_2374-8265.10994-s001.zip › C. Simulation Case 3 Template.docx]

| Appendix C: Simulation Case #3 Template  SIMULATION CASE TITLE: Scenario #3: Tracheostomy and ventilator-dependent patient with mucus plugging with progression to cardiopulmonary arrest  AUTHORS: Khan EK, MD; Lockspeiser TM, MD; Liptzin DR, MD, MS; Baker CD, MD  **LEARNER AUDIENCE:** Pediatric Resident Physicians | |
| --- | --- |
| **PATIENT NAME:** Baby Ruth  **PATIENT AGE:** 10 months  **CHIEF COMPLAINT:** High-pressure ventilator alarm  **PHYSICAL SETTING:** In classroom, empty patient room, or simulation center. Mannequin with tracheostomy in place, connected to ventilator with “high pressure” alarm.  *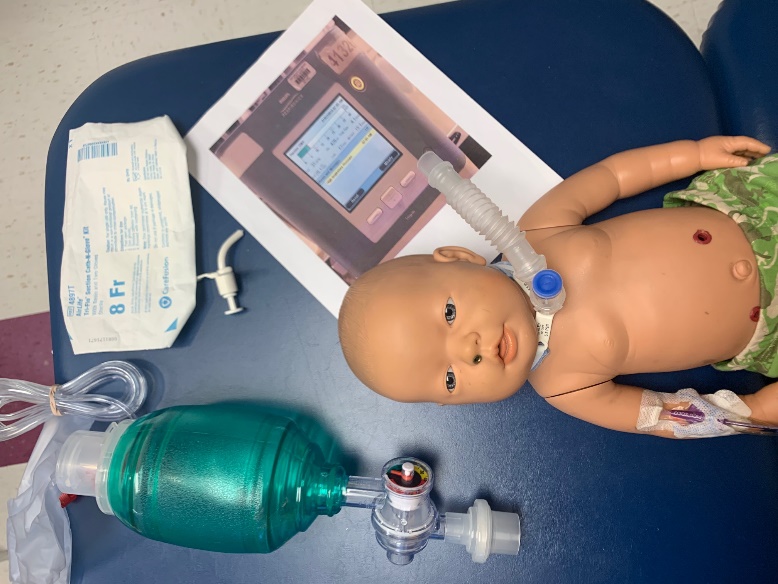*  *Image Citation: Author Owned* | |
| **Brief narrative description of case** | This scenario involves a tracheostomy and ventilator dependent patient who experiences tracheostomy tube obstruction requiring suctioning and changing the tracheostomy tube. The final required intervention in this scenario is to activate a Code Blue response and begin cardiopulmonary resuscitation in response to cardiorespiratory arrest. |
| **Primary Learning Objectives** | 1. Identify when the child’s status is deteriorating based on color change, neurological response, coughing, work of breathing, and ventilator alarm status. 2. Demonstrate correct steps to alleviate a plugged tracheostomy tube by suctioning and changing the tracheostomy tube. 3. Demonstrate emergency action steps including calling Code Blue and providing adequate cardiopulmonary resuscitation. |
| **Critical Actions** | Stress importance of checking stoma for tracheostomy tube placement, equipment connections, suctioning, “when in doubt, change it out”, and rapid initiation of chest compressions in the bradycardic patient |
| **Learner Preparation or Prework** | None |

| Initial Presentation: “It is lunchtime and you are headed to grab some food. You walk past your favorite patient’s room and hear the ventilator alarm.” | | | |
| --- | --- | --- | --- |
| **Initial vital signs** | See Appendix D Case Scenario Visual Cards: vital signs 3.1  HR 170/min  BP 90/50  RR 45/min  Sat 89% on RA | | |
| **Overall Setting and Appearance** | See Appendix D Case Scenario Visual Cards: vital signs 3.1  Dusky  Anxious  Increased WOB  Slightly diminished aeration with crackles and rhonchi | | |
| **Confederates (e.g., standardized participants) and their roles in the room at case start** | Facilitator: to guide learner through case stages  Learner(s): 1-3 resident physicians, each taking turns “leading” each scenario while others provided back-up as “helpers” when called upon by lead resident | | |
| **HPI** | This is a 10-month-old former 26-week premature infant with tracheostomy and ventilator dependence. She has been in usual state of health, awaiting discharge criteria of educating home providers. | | |
| **Past Medical/Surgical History** | **Medications** | **Allergies** | **Family History** |
| 26-week prematurity with uncomplicated NICU course, mild well controlled pulmonary hypertension, mild reflux, gastrostomy tube dependence | Sildenafil | None | None |
| Physical Examination - See Appendix D Case Scenario Visual Cards (intentionally limited) | | | |

| Instructor Notes - Changes and CASE Branch Points | | | | |  |  |
| --- | --- | --- | --- | --- | --- | --- |
| STAGE | | VITAL SIGNS 3.1 | PHYSICAL EXAM | PARTICIPANTS’ REQUIRED ACTS | NOTES TO OPERATOR | |
| Initial assessment | | HR 170/min  BP 90/50  RR 45/min  Sat 89% on 2LPM | Dusky  Anxious  Slightly diminished aeration with crackles and rhonchi | Check airway/trach/ventilator connections.   1. Does not perform correctly 2. Performs correctly with guidance or prompting 3. Performs correctly without assistance *[visualizes trach entering stoma, checks ventilator connection]* | Home ventilator, simulate high pressuring  Suction available  Tracheostomy to have thick secretions with a “plug” | |
| STAGE | | VITAL SIGNS 3.2 | PHYSICAL EXAM | PARTICIPANTS’ REQUIRED ACTS | NOTES TO OPERATOR | |
| Intervention: Suctioning | | HR 175/min  BP 80/40  RR 60/min  Sat 82% | Blue  Anxious  Worsening aeration, severely diminished | Responds to high vent alarm by suctioning and/or changing trach.   1. Does not perform correctly 2. Performs correctly with guidance or prompting 3. Performs correctly without assistance *[suctions with sterile technique]* | Resident suctions trach, may try saline.  If resident moves to any other step, prompt: Would you like to try suctioning before moving on?  Educator’s Prompt: “You are unable to pass suction catheter.” | |
| STAGE | | VITAL SIGNS 3.3 | PHYSICAL EXAM | PARTICIPANTS’ REQUIRED ACTS | NOTES TO OPERATOR | |
| Intervention: Change Trach | | HR 100/min  BP 60/30  RR 20/min  Sat 75% | Lethargic | Changes trach.   1. Does not perform correctly 2. Performs correctly with guidance or prompting 3. Performs correctly without assistance *[changes trach with assistance from “helper”]* | Resident changes the trach. As Resident changes trach, child becomes unresponsive.  If resident moves to any other step or unsure of what to do, prompt: Do you believe the airway is fully intact? And/or Would you like to try changing the tracheostomy tube? | |
| STAGE | | VITAL SIGNS 3.4 | PHYSICAL EXAM | PARTICIPANTS’ REQUIRED ACTS | NOTES TO OPERATOR | |
| Intervention:  Activate Code Blue | | HR 20/min  BP Unable to Obtain  RR 0  Sat 50% | Eyes closed  Unresponsive | Activates Code Blue response.   1. Does not perform correctly 2. Performs correctly with guidance or prompting/delay 3. Performs correctly without assistance *[activate code blue response]* | Resident activates Code Blue response  If resident moves to any other step, prompt: Would you like to call for help? | |
| STAGE | | VITAL SIGNS 3.4 | PHYSICAL EXAM | PARTICIPANTS’ REQUIRED ACTS | NOTES TO OPERATOR | |
| Intervention: Begin CPR | | HR 20/min  BP Unable to Obtain  RR 0  Sat 50% | Eyes closed  Unresponsive | Responds to cardiopulmonary arrest.   1. Does not perform correctly 2. Performs correctly with guidance or prompting/delay 3. Performs correctly without assistance *[call for help, begin chest compression/bagging]* | Calls for help, begins chest compressions while alternative resident begins bag-trach ventilation with 100% FiO2. No alternating ratio needed for compressions and rescue breaths given invasive airway. | |
| STAGE | | VITAL SIGNS 3.5 | PHYSICAL EXAM | PARTICIPANTS’ REQUIRED ACTS | NOTES TO OPERATOR | |
| Child recovers | | HR 140/min  BP 80/40  RR 20 (assisted)  Sat 85% | Sleepy but now awake  Good aeration with fine crackles | Resident stops CPR.  Places child back on ventilator. | End of simulation, enter debriefing. | |

Debriefing:

1. Description: Reinforce safe learning environment, focus on what happened

Example questions: So, what happened? How did that go?

1. Analysis: Analyze and explore what happened in detail

Example questions: What do you think was going on when the patient had high pressure alarms? Why did the patient decompensate?

1. Application: Move from specifics of this case to general practice

Example questions: What else could have been going on? Why did you delay starting chest compressions?

**Ideal Scenario Flow:**

The learner enters patient room and provides initial assessment - visualizes trach entering stoma, checks ventilator connection, notes high ventilator alarm notification. The learner recognizes tracheostomy tube obstruction and suctions with sterile technique. Patient continues to worsen despite suctioning, therefore learner changes tracheostomy tube with assistance from “helper”. When the patient becomes unresponsive with bradycardia, apnea, and hypotension, learner activates code blue response, calls for help, and begins cardiopulmonary resuscitation (CPR) with chest compressions and bag-trach ventilation. Patient improves and begins to recover; resident stops CPR.

**Anticipated Management Mistakes: (see above “Notes to Operator”)**

1. Failure to visualize tracheostomy tube entering stoma: Many learners will not visualize the tube entering the stoma and often needed to be reminded of this key step.
2. Delay in initiating chest compressions: Occasionally learners would be slow to initiate chest compressions or bag-trach ventilation. We often addressed this in debriefing as listed in example questions above.
