# Supplementary material for: “When in Doubt, Change It out”: A Case-Based Simulation for Pediatric Residents Caring for Hospitalized Tracheostomy-Dependent Children
Source: MedEdPORTAL. 2020 Oct 1;16:10994. doi: 10.15766/mep_2374-8265.10994 (PMC7528672; doi:10.15766/mep_2374-8265.10994)
Supplement: Supplementary file 1 — Simulation Case 1 Template.docxSimulation Case 2 Template.docxSimulation Case 3 Template.docxAssessment Score Sheet.docxCase Scenario Visual Cards.docxSimulation Feedback Tool.docx [file mep_2374-8265.10994-s001.zip › D. Assessment Score Sheet.docx]

**Scenario #1: Trach/vent-dependent patient with vent malfunction, trach complications**

| **STAGE 1.1** | Initial Assessment |
| --- | --- |
| **Skill** | *Visualizes trach entering Stoma, checks ventilator connection* |
| **Score** | **0 - 1 - 2** |
|  | |
| **STAGE 1.1** | Intervention – Reconnect Ventilator |
| **Skill** | *Establishes ventilator connection* |
| **Score** | **0 - 1 - 2** |
|  | |
| **STAGE 1.2** | Intervention – Suctioning |
| **Skill** | *Suctions with sterile technique* |
| **Score** | **0 - 1 - 2** |
|  | |
| **STAGE 1.2** | Intervention – Change Tracheostomy Tube |
| **Skill** | *Changes to new trach with “helper”* |
| **Score** | **0 - 1 - 2** |
|  | |
| **STAGE 1.3** | Intervention – Increase Oxygen |
| **Skill** | *Adds supplemental O2* |
| **Score** | **0 - 1 - 2** |
|  | |
| **STAGE 1.4** | SIM over |
| **Total Score** |  |

**Scenario #2: Trach-dependent patient with accidental decannulation**

| **STAGE 2.1** | Initial Assessment |
| --- | --- |
| **Skill** | *Visualizes trach entering stoma* |
| **Score** | **0 - 1 - 2** |
|  | |
| **STAGE 2.2** | Intervention – Replaces decannulated trach |
| **Skill** | *Replaces dislodged trach* |
| **Score** | **0 - 1 - 2** |
|  | |
| **STAGE 2.2** | Intervention – Suctioning |
| **Skill** | *Suctions with sterile technique* |
| **Score** | **0 - 1 - 2** |
|  | |
| **STAGE 2.2** | Intervention – Change Tracheostomy Tube |
| **Skill** | *Changes to new trach with “helper”* |
| **Score** | **0 - 1 - 2** |
|  | |
| **STAGE 2.3** | Intervention – Bag-Trach Ventilation |
| **Skill** | *Bags through trach, may ask for advice on PEEP settings* |
| **Score** | **0 - 1 - 2** |
|  | |
| **STAGE 2.4** | SIM over |
| **Total Score** |  |

**Scenario #3: Trach/vent-dependent patient, mucus plugging, progression to CPR**

| **STAGE 3.1** | Initial Assessment |
| --- | --- |
| **Skill** | *Visualizes trach entering stoma, checks ventilator connection* |
| **Score** | **0 - 1 - 2** |
|  | |
| **STAGE 3.2** | Intervention – Suctioning |
| **Skill** | *Suctions with sterile technique* |
| **Score** | **0 - 1 - 2** |
|  | |
| **STAGE 3.3** | Intervention – Change Tracheostomy Tube |
| **Skill** | *Changes trach with assistance from “helper”* |
| **Score** | **0 - 1 - 2** |
|  | |
| **STAGE 3.4** | Intervention – Activate Code Blue |
| **Skill** | *Activates code blue response* |
| **Score** | **0 - 1 - 2** |
|  | |
| **STAGE 3.4** | Intervention – Begin CPR |
| **Skill** | *Cal for help, begin chest compression/bagging* |
| **Score** | **0 - 1 - 2** |
|  | |
| **STAGE 3.5** | SIM over |
| **Total Score** |  |
