# Supplementary material for: “When in Doubt, Change It out”: A Case-Based Simulation for Pediatric Residents Caring for Hospitalized Tracheostomy-Dependent Children
Source: MedEdPORTAL. 2020 Oct 1;16:10994. doi: 10.15766/mep_2374-8265.10994 (PMC7528672; doi:10.15766/mep_2374-8265.10994)
Supplement: Supplementary file 1 — Simulation Case 1 Template.docxSimulation Case 2 Template.docxSimulation Case 3 Template.docxAssessment Score Sheet.docxCase Scenario Visual Cards.docxSimulation Feedback Tool.docx [file mep_2374-8265.10994-s001.zip › E. Case Scenario Visual Cards.docx]

**Tracheostomy Simulation Visual Assistance for Learners: Vital Signs**

**Suggested Use:** Print and laminate individual vital sign cards and physical exam prompts. These may be sequentially revealed to learners as cases progress.

| **VITAL SIGNS 1.1** | **PHYSICAL EXAM** |
| --- | --- |
| HR 170/min  BP 90/50  RR 45/min  Sat 89% on RA | Dusky  Anxious  Increased WOB  Slightly diminished aeration with crackles and rhonchi |

| **VITAL SIGNS 1.2** | **PHYSICAL EXAM** |
| --- | --- |
| HR 175/min  BP 80/40  RR 60/min  Sat 82% on RA | Blue  Anxious  Still with diminished aeration |

| **VITAL SIGNS 1.3** | **PHYSICAL EXAM** |
| --- | --- |
| HR 120/min  BP 90/50  RR 40/min  Sat 82% on RA | Awake, alert  Breathing comfortably with good aeration and good chest rise, symmetrical exam |

| **VITAL SIGNS 1.4** | **PHYSICAL EXAM** |
| --- | --- |
| HR 110/min  BP 90/50  RR 30/min  Sat 95% on 2LPM | Happy and playful  Back to baseline |

| **VITAL SIGNS 2.2** | **PHYSICAL EXAM** |
| --- | --- |
| HR 175/min  BP 100/60  RR 60/min  Sat 80% on 2LPM | Blue  Anxious  Eyes open  Increased WOB  Extremely diminished aeration |

| **VITAL SIGNS 2.1** | **PHYSICAL EXAM** |
| --- | --- |
| HR 170/min  BP 100/60  RR 55/min  Sat 83% on 2LPM | Pale/Dusky  Anxious  Eyes open  Increased WOB  Extremely diminished aeration |

| **VITAL SIGNS 2.4** | **PHYSICAL EXAM** |
| --- | --- |
| HR 140/min  BP 100/60  RR 40  Sat 97% | Improved color  Improved aeration |

| **VITAL SIGNS 2.3** | **PHYSICAL EXAM** |
| --- | --- |
| HR 160/min  BP 90/55  RR 50/min  Sat 80% on 2LPM | Blue  Anxious  Eyes open  Increased WOB  Somewhat improved aeration but still diminished |

| **VITAL SIGNS 3.1** | **PHYSICAL EXAM** |
| --- | --- |
| HR 170/min  BP 90/50  RR 45/min  Sat 89% on 2LPM | Dusky  Anxious  Slightly diminished aeration with crackles and rhonchi |

| **VITAL SIGNS 3.2** | **PHYSICAL EXAM** |
| --- | --- |
| HR 175/min  BP 80/40  RR 60/min  Sat 82% | Blue  Anxious  Worsening aeration, severely diminished |

| **VITAL SIGNS 3.3** | **PHYSICAL EXAM** |
| --- | --- |
| HR 100/min  BP 60/30  RR 20/min  Sat 75% | Lethargic |

| **VITAL SIGNS 3.4** | **PHYSICAL EXAM** |
| --- | --- |
| HR 20/min  BP UTA  RR UTA  Sat 50% | Eyes closed  Unresponsive |

| **VITAL SIGNS 3.5** | **PHYSICAL EXAM** |
| --- | --- |
| HR 140/min  BP 80/40  RR 20 (assisted)  Sat 85% | Sleepy but now awake  Good aeration with fine crackles |
