# Supplementary material for: “When in Doubt, Change It out”: A Case-Based Simulation for Pediatric Residents Caring for Hospitalized Tracheostomy-Dependent Children
Source: MedEdPORTAL. 2020 Oct 1;16:10994. doi: 10.15766/mep_2374-8265.10994 (PMC7528672; doi:10.15766/mep_2374-8265.10994)
Supplement: Supplementary file 1 — Simulation Case 1 Template.docxSimulation Case 2 Template.docxSimulation Case 3 Template.docxAssessment Score Sheet.docxCase Scenario Visual Cards.docxSimulation Feedback Tool.docx [file mep_2374-8265.10994-s001.zip › F. Simulation Feedback Tool.docx]

**SIMULATION FEEDBACK TOOL**

**Suggested Use:** Send anonymously to learners via email following completion of simulation, if desired.

**Question 1:** How helpful was this simulation experience in expanding your ability to care for the tracheostomy and/or ventilator dependent child?

**Answer Choices:**

Extremely helpful

Very helpful

Somewhat helpful

Not so helpful

Not at all helpful

**Question 2:** Do you have any feedback regarding this simulation experience?

**Answer Choices:** (open ended response)
