# Supplementary material for: “When in Doubt, Change It out”: A Case-Based Simulation for Pediatric Residents Caring for Hospitalized Tracheostomy-Dependent Children
Source: MedEdPORTAL. 2020 Oct 1;16:10994. doi: 10.15766/mep_2374-8265.10994 (PMC7528672; doi:10.15766/mep_2374-8265.10994)
Supplement: Supplementary file 1 — Simulation Case 1 Template.docxSimulation Case 2 Template.docxSimulation Case 3 Template.docxAssessment Score Sheet.docxCase Scenario Visual Cards.docxSimulation Feedback Tool.docx [file mep_2374-8265.10994-s001.zip › _Educational_Summary_Report_10994.pdf]

MedEdPORTAL® The AAMC Journal of Teaching and Learning Resources

Original Publication Open Access

"When in Doubt, Change It out": A Case-Based Simulation for Pediatric Residents Caring for Hospitalized Tracheostomy-Dependent Children

Erin K. Khan, MD*, Tai M. Lockspeiser, MD, MHPE, Deborah R. Liptzin, MD, MS, Maxene Meier, MS, Christopher D. Baker, MD

*Corresponding Author: ekhan@cmh.edu

Abstract

Introduction: Caring for technology-dependent children, such as those with tracheostomy and ventilator dependence, can be new and frightening for pediatric residents. Education about emergencies in this patient population is important because these children are at risk for in-hospital complications. Safe care of the tracheostomy-dependent child requires the ability to recognize common complications, such as tracheostomy tube obstruction or decannulation, and intervene appropriately by suctioning and/or replacing the tracheostomy tube. This simulation-based curriculum teaches learners to identify and practice the management of these tracheostomy tube complications through low-fidelity simulation exercises. **Methods:** We created a simulation session with three cases reflecting in-hospital scenarios encountered by resident physicians caring for tracheostomy-dependent children in the inpatient setting. The simulation scenario, simulation environment preparation, materials list, and debriefing outline are provided for the instructor for each simulation case. Validity evidence for the assessment tool was obtained by calculating the interrater reliability of two different raters. Resident feedback was obtained through anonymous surveys. **Results:** Twelve pediatric senior residents completed the experience. It received overwhelmingly positive feedback on learner evaluation forms, with 90% finding the experience very or extremely helpful. The intraclass correlation coefficient of interrater reliability for our assessment tool was 0.93. **Discussion:** The simulation was well received by residents. The interrater reliability was acceptable. This low-fidelity simulation exercise can easily be executed with minimal materials or instructor training. High-yield, just-in-time training with postcase debriefing is key to the simulation's success.

Keywords
Tracheostomy, Simulation, Pediatrics, Low Fidelity, Pulmonology, Clinical Reasoning/Diagnostic Reasoning, Pediatric Critical Care Medicine, Pediatric Pulmonology, Case-Based Learning

Educational Objectives

By the end of this simulation, learners will be able to:

1. Suction a tracheostomy tube.
2. Change a tracheostomy tube.
3. Demonstrate an appropriate response to a decompensating patient with a tracheostomy tube with or without a ventilator in a simulated session, by doing one or more of the following: (a) responding to common ventilator issues such as low-pressure alarm by evaluating the ventilator system and tubing, (b) responding to tracheostomy tube obstruction by suctioning and/or changing the tracheostomy tube, (c) responding to hypoxemia by increasing oxygen supplementation, and/or (d) activating code blue alarm in response to cardiorespiratory arrest in the patient.

Citation:
Khan EK, Lockspeiser TM, Liptzin DR, Meier M, Baker CD. "When in doubt, change it out": a case-based simulation for pediatric residents caring for hospitalized tracheostomy-dependent children. *MedEdPORTAL*. 2020;16:10994.
https://doi.org/10.15766/mep_2374-8265.10994

Introduction

Children with tracheostomy and ventilator requirements are at risk for tracheostomy-related complications.[1-5] Appropriate evaluation of the acutely decompensating patient with tracheostomy dependence challenges the normal Pediatric Advanced Life Support algorithm of circulation-airway-breathing.[6] The common complications in patients with tracheostomy dependence are primarily airway related and thus warrant following the more traditional airway-breathing-circulation algorithm. These airway-related complications include accidental decannulation as well as tracheostomy tube obstruction, which may be due to mucus plugging, increased secretions during infection, granulomas, bleeding into the airway, or improper tube

Copyright © 2020 Khan et al. This is an open-access publication distributed under the terms of the Creative Commons Attribution license.

position (against the tracheal wall).[1-4] One must rapidly recognize and appropriately intervene when airway-related complications occur to provide safe care and prevent harm.[2,7,8]

Regardless of the etiology of an acute decompensation event in the tracheostomy-dependent child, primary management consists of establishing a patent airway by suctioning and/or replacing the tracheostomy tube.[8] Many centers, including ours, have patients with tracheostomy and ventilator requirements on the pediatric wards where residents provide care. Routine tracheostomy tube care and skills are not regularly taught to pediatric residents.[9,10] Inadequate education of providers caring for patients with tracheostomies has resulted in increased adverse events in these patients.[7,11] Therefore, resident physicians tasked with caring for patients with tracheostomy and ventilator requirements require specialized training to safely and confidently perform the appropriate steps for tracheostomy tube suctioning and replacement.[7,11-13]

Simulations have long been shown to provide a safe space for learners to practice unfamiliar skills.[14] Simulation has been used in the tracheostomy patient population to create realistic clinical scenarios that allow learners to seek feedback, reflect, and learn with no risk to the patient.[9,15-17] However, there are no standardized simulation curricula for pediatric residents caring for the inpatient tracheostomy- and/or ventilator-dependent patient. While simulation is largely a formative process, meant to build skills in learners, it also can be used as an assessment tool to evaluate learner competence and the strength of teaching curricula.[18]

This simulation-based experience provides a framework to educate, assess, and debrief learners caring for the child with tracheostomy and ventilator requirements. The curriculum uses low-fidelity simulation with little to no specialized equipment or facilitator training required. Through identification of tracheostomy tube complications and skills practice during low-fidelity simulation exercises, resident physicians are better equipped to care for the hospitalized patient with tracheostomy and ventilator requirements.

Methods

Development

In 2018, Thrasher and colleagues published two high-fidelity simulation scenarios for use in training family caregivers of the tracheostomy- and ventilator-dependent child for independent care at home.[15] More recently, the team developed a third high-fidelity scenario to simulate tracheostomy tube decannulation in the tracheostomy-dependent patient. With permission, we adjusted these three scenarios to reflect inpatient experiences frequently encountered by pediatrics residents on our pulmonary unit. No prerequisite knowledge was required.

Equipment/Environment

The cases were easily performed in a low-fidelity simulation environment. The environment can be in a classroom, bedside in an empty patient room, or in a simulation center. For ease of administration, we chose not to require an actual home ventilator for the simulation and instead used visual cards to demonstrate common ventilator alarms of high pressure and low pressure/circuit disconnect. Simulation facilitators can easily create laminated photographs of the home ventilator used in their program to display the desired alarms.

Supplies needed (Figure 1) included the following:

- Low-fidelity simulation mannequin—we initially used an inexpensive stuffed animal, simply modified to have a tracheostomy stoma (Figure 2).
- Tracheostomy tubes—one main tube and one backup/replacement tube.
- Tracheostomy ties.
- Suction catheter.
- Bag ventilation equipment.
- Ventilator connection tubing.
- Ventilator alarm notification visual cards—to simulate high- and low-pressure alarms.
- Case scenario visual cards (Appendix E)—to simulate vital sign changes.
- Simulation facilitator guide and assessment tool (Appendices A-D).
- Feedback tool (Appendix F).

Personnel

This simulation requires minimal personnel support:

- Facilitator (one)—skilled in the care of the tracheostomy-dependent child.
- Learners/participants (one to three).
- Simulation mannequin with tracheostomy capabilities (Figures 1 and 2).

Implementation

After we had received approval from the institutional review board and obtained informed consent from participants, a subset of second- and third-year pediatric residents at our center completed this simulation experience from June to August 2019 during their inpatient pulmonary month or at an academic half-day experience focused on pulmonary medicine. Scenarios were

Copyright © 2020 Khan et al. This is an open-access publication distributed under the terms of the Creative Commons Attribution license.

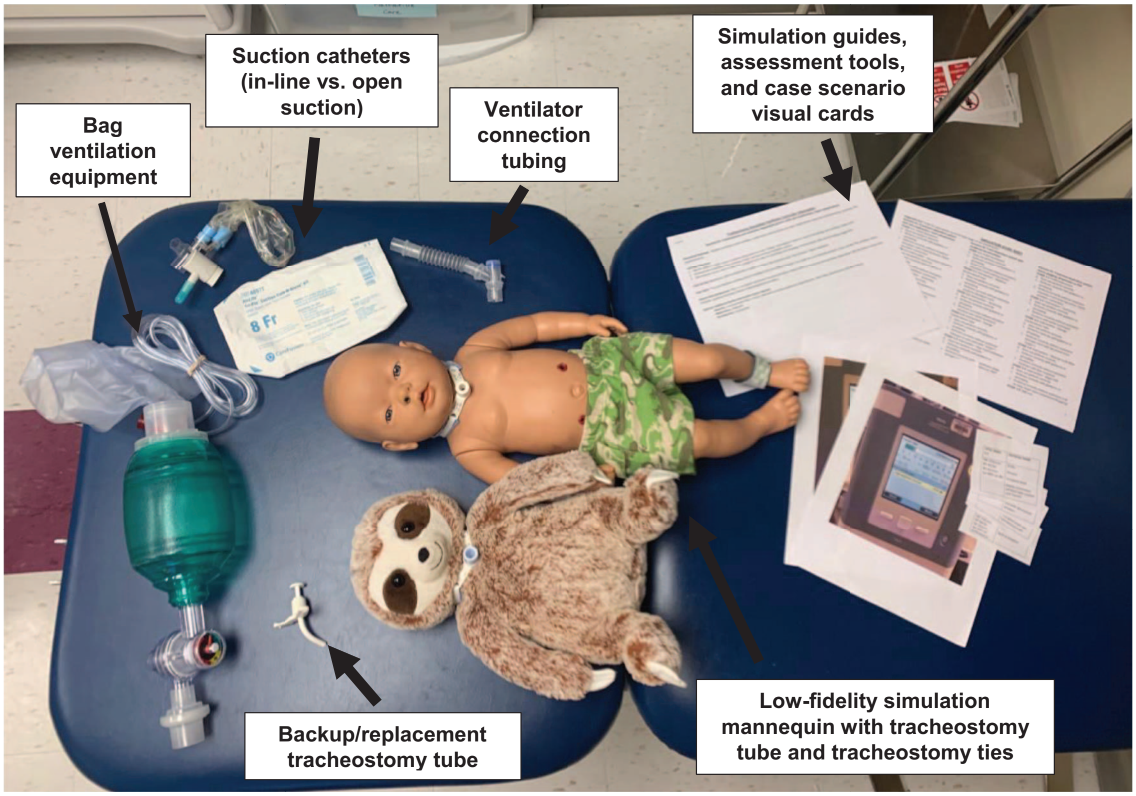

Figure 1. Supplies needed.

performed in a classroom setting with low-fidelity simulation equipment. Facilitator guides (Appendices A-D) described case setup requirements.

Appendix A contains Simulation Case Scenario 1 facilitator curricular information. This scenario involved a tracheostomy- and ventilator-dependent patient who experienced ventilator malfunction in the form of ventilator disconnect, followed by tracheostomy complications of tube obstruction requiring suctioning and changing the tracheostomy tube. The final required intervention in this scenario was to increase oxygen in response to hypoxemia.

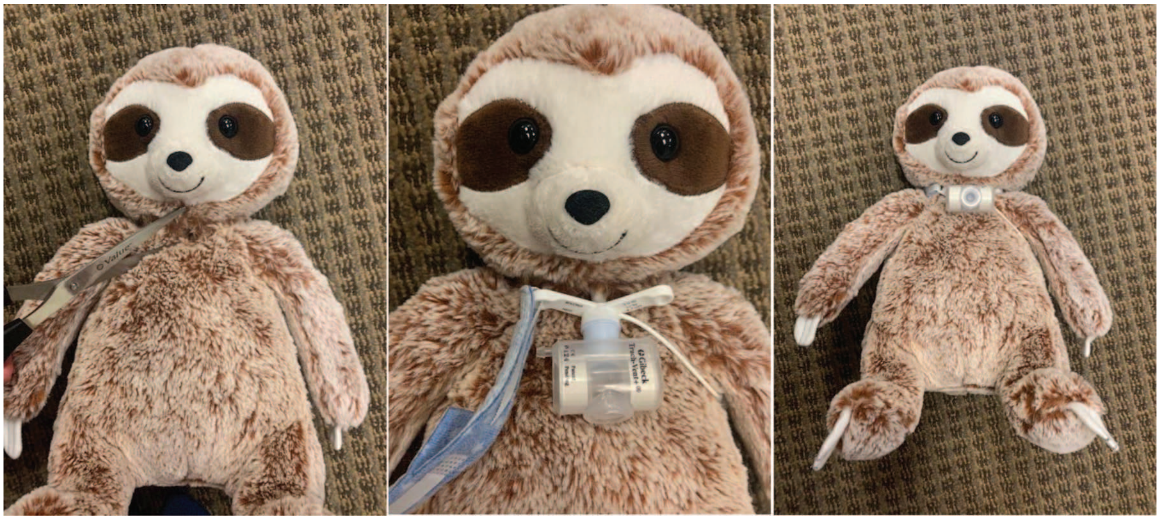

Figure 2. Demonstration of simple modification of a stuffed animal to accommodate low-fidelity tracheostomy simulation.

Copyright © 2020 Khan et al. This is an open-access publication distributed under the terms of the Creative Commons Attribution license.

Appendix B contains Simulation Case Scenario 2 facilitator curricular information. This scenario involved a tracheostomy-dependent patient who experienced accidental decannulation of the tracheostomy tube requiring replacement of the tube, suctioning, and changing the tracheostomy tube. The final required intervention in this scenario was to provide bag-trach ventilation in response to poor respiratory effort.

Appendix C contains Simulation Case Scenario 3 facilitator curricular information. This scenario involved a tracheostomy- and ventilator-dependent patient who experienced tracheostomy tube obstruction requiring suctioning and changing the tracheostomy tube. The final required intervention in this scenario was to activate a code blue response and begin cardiopulmonary resuscitation in response to cardiorespiratory arrest.

Appendix D provides an easy-to-use assessment score sheet for each scenario. Appendix E contains case scenario visual cards, including vital sign data and physical exam information for each stage of the three scenarios. These visual cards can be printed and laminated for repeat use. Appendix F details an optional end-of-experience learner feedback tool.

Prior to starting each scenario, the facilitator set expectations with all learners. One resident would take the lead in each simulation scenario, while the remaining learners observed until called upon to be a helper by the main participant. Residents rotated roles during different scenarios. The simulation was meant to be formative, allowing each learner to have hands-on time while troubleshooting common acute scenarios. Each scenario took approximately 20 minutes to complete, including time for structured debriefing.

Assessment

We also developed an assessment tool to be used during the simulations (Appendix D). This tool listed required steps for the learner to complete for each case scenario of a decompensating child with tracheostomy and/or ventilator dependence, including items such as visualizing the tracheostomy tube entering the stoma, assessing the ventilator connections, recognizing common complications, and intervening by suctioning and/or replacing the tracheostomy tube. With each key step, the learner was scored on a 0-2 scale. If the learner performed the step without assistance, they received a maximum score of 2. If the learner required assistance to complete, they were scored 1 on that step. If the learner performed incorrectly or could not perform even with assistance, they received a 0 for that step. Each case had five key steps, with a maximum total score of 10.

Each resident was given a single simulation case and was scored using the assessment tool by two raters. A total of three raters participated in the scoring, including a primary rater and one of two secondary raters for each case. Raters included pulmonology-trained physicians and nurses with experience and expertise in caring for the tracheostomy-dependent child. Prior to scoring the participants, raters reviewed the assessment tool together to calibrate the rating scale. Interrater reliability was calculated using the intraclass correlation coefficient (ICC). Resident feedback was obtained through anonymous online surveys following the simulation.

Debriefing

Debriefing was held after every scenario using the Debrief Diamond structure (Appendices A-C) created by Jaye, Thomas, and Reedy.[19] The components of the Debrief Diamond—description, analysis, and application—facilitated an open discussion following simulation scenarios. Description started by reinforcing a safe learning environment and asking the learner to evaluate what took place during the scenario. (Example: "How do you think that went?") Analysis allowed the team to assess the specifics of the simulation flow. This step let the facilitator probe for understanding of clinical findings and educate, reinforce, or redirect clinical decision-making. (Example: "What do you think was going on when the patient's ventilator had high-pressure alarms?") A majority of the debriefing time was spent in analysis, with the facilitator promoting learner self-reflection rather than simply providing judgment of the participants' actions.[20] Lastly, application focused on moving forward to reinforce learning by applying the principles discussed in the simulation to real life. (Example: "What would you have done if the patient did not respond to tracheostomy change?")

Results

The simulation scenarios were completed by 12 pediatric senior residents, a subset of residents from our training program who were available and consented to participate during previously scheduled educational activity sessions throughout the 3-month study period. All 12 residents were scored by two raters. The assessment tool demonstrated good interrater reliability with an ICC of 0.93. Ten of 12 participants (83%) completed the online postsession evaluation (Appendix F). Ninety percent of respondents found the simulation very or extremely helpful in expanding their ability to care for the tracheostomy-dependent patient.

Qualitative analysis of open-ended survey responses was uniformly positive. Residents appreciated the simulation

Copyright © 2020 Khan et al. This is an open-access publication distributed under the terms of the Creative Commons Attribution license.

experience and felt that it improved their clinical care. For example, one resident stated, “I felt so much more confident and capable in taking care of kids with trachs after this workshop,” and another succinctly commented, “I loved it and felt more prepared because of it.” Residents also specifically commented on the benefit of hands-on training: “The hands-on nature of the simulation was excellent... Much more effective than a lecture on how to change or troubleshoot a trach.”

Discussion

Caring for the tracheostomy-dependent patient requires a unique skill set not frequently taught to pediatric resident physicians. Patient care–related actions such as suctioning and changing a tracheostomy tube are unable to be adequately taught or assessed by educational means such as lectures or handout-based learning materials. One must instead utilize active learning strategies to teach such skills. Our learners especially enjoyed the hands-on nature of this learning activity.

Simulation is a cornerstone of health professional education, creating a realistic environment in which to practice skills with no harm to the patient. This benefit is particularly relevant to the high-risk population of tracheostomy-dependent patients. However, an AAMC survey targeting the use of simulation in medical education found that only about 30% of residents training in teaching hospitals reported using simulation in this patient population.[14] We created this low-fidelity simulation experience to help pediatric residents recognize and respond to common complications of the hospitalized tracheostomy-dependent patient. The acceptable interrater reliability of our assessment tool, even with minimal rater training, provides initial validity evidence for using this simulation as a means of learner assessment as well. Feedback from our learners was overwhelmingly positive.

While the specific case scenarios here are geared toward the pediatric patient, case stems and visual cards could easily be revised to reflect the adult tracheostomy-dependent patient. A strong benefit of our curriculum is its use of a low-fidelity, minimal-cost simulation environment. Our learners were able to adequately and appropriately demonstrate the desired skills on our low-fidelity mannequin. This simulation can be readily recreated with or without need for specialized simulation environments or complex equipment, allowing the exercise to be performed easily in any environment, including a classroom, an empty patient room, or a specialized simulation center.

Many trainees may not have previously cared for patients with acute tracheostomy-related decompensations. If the learner is failing to appropriately intervene during the simulation, it is preferable to pause and redirect rather than allow the mannequin to severely decompensate or expire. Keeping in line with cognitive load theory,[21] assistance should be provided to the learner to prevent excessive struggling and a negative learning experience during the simulation. We approached this simulation as a formative process. However, our novel assessment tool could allow for a summative assessment of learners as well.

Debriefing, when done well, is instrumental in providing effective education and a positive learning experience for trainees during simulation scenarios. An approach of debriefing with good judgment allows the facilitator to investigate the simulation participants’ motives behind certain actions, or lack thereof, rather than placing judgment on the learners.[20] The Debriefing Diamond provides a structured way to debrief with learners following simulation scenarios that is simple, effective, and well received.[19] This style of debriefing, in combination with the structured assessment tool, allows the simulation facilitator to ensure teaching is effective.

Limitations of our study include those commonly found in simulation education, including difficulties in creating a true-to-life scenario using low-fidelity simulation equipment. Using a high-fidelity mannequin would have provided a more realistic experience for learners, but such a curriculum would be prohibitively difficult for many training programs to implement. We also recognize our small sample size. As mentioned above, we approached this simulation as a formative process and intentionally scored enough learners to be able to adequately calculate the interrater reliability. Having a larger group of pediatric residents complete the simulation training will help address this limitation, as described below.

Since completion of this initial study, we have continued to use the simulation curriculum and assessment tool as a part of a larger ongoing educational program for all 35 of our second-year pediatric residents each year. This program, the Pediatric Resident Education in Pulmonary (PREP) Boot Camp, is a half-day session held monthly on the residents’ first day of their inpatient pediatric pulmonary rotation. PREP Boot Camp includes multiple high-yield interactive lectures and hands-on sessions focusing on preparing residents to care for complex pulmonary inpatients, including those with cystic fibrosis and asthma in addition to tracheostomy and ventilator dependence.[22] This ongoing experience further adds to the validity evidence for this curriculum and is the focus of planned future publications. In addition to PREP Boot Camp, other future directions include

Copyright © 2020 Khan et al. This is an open-access publication distributed under the terms of the Creative Commons Attribution license.

assessing both short- and long-term patient outcomes as a result of our enhanced educational program.

Overall, this simulation session has proven to be a useful and cost-effective way for pediatric trainees to learn to troubleshoot common tracheostomy-related complications. Such training adds to both the learning experience of trainees and the safety of high-risk technology-dependent children.

Appendices

A. Simulation Case 1 Template.docx

B. Simulation Case 2 Template.docx

C. Simulation Case 3 Template.docx

D. Assessment Score Sheet.docx

E. Case Scenario Visual Cards.docx

F. Simulation Feedback Tool.docx

All appendices are peer reviewed as integral parts of the Original Publication.

Erin K. Khan, MD: Fellow, Department of Pediatrics, Section of Pulmonology and Sleep Medicine, University of Colorado School of Medicine; ORCID: https://orcid.org/0000-0001-6629-6164

Tai M. Lockspeiser, MD, MHPE: Associate Professor, Department of Pediatrics, University of Colorado School of Medicine; Assistant Dean of Medical Education, University of Colorado School of Medicine; ORCID: https://orcid.org/0000-0002-1633-4860

Deborah R. Liptzin, MD, MS: Assistant Professor, Department of Pediatrics, Section of Pulmonology and Sleep Medicine, University of Colorado School of Medicine; ORCID: https://orcid.org/0000-0002-3667-1856

Maxene Meier, MS: Research Instructor, Department of Pediatrics, University of Colorado School of Medicine; ORCID: https://orcid.org/0000-0003-3342-1816

Christopher D. Baker, MD: Associate Professor, Department of Pediatrics, Section of Pulmonology and Sleep Medicine, University of Colorado School of Medicine; Director of Ventilator Care Program, University of Colorado School of Medicine; ORCID: https://orcid.org/0000-0002-1171-563X

Disclosures
None to report.

Funding/Support
None to report.

Ethical Approval
The University of Colorado Institutional Review Board approved this study.

References

1. Dal'Astra AP, Quirino AV, Caixêta JA, Avelino MA. Tracheostomy in childhood: review of the literature on complications and mortality over the last three decades. *Braz J Otorhinolaryngol*. 2017;83(2):207-214. https://doi.org/10.1016/j.bjorl.2016.04.005
2. Maunsell R, Avelino M, Caixeta Alves J, et al. Revealing the needs of children with tracheostomies. *Eur Ann Otorhinolaryngol Head Neck Dis*. 2018;135(5)(suppl):S93-S97. https://doi.org/10.1016/j.anorl.2018.07.007
3. Watters KF. Tracheostomy in infants and children. *Respir Care*. 2017;62(6):799-825. https://doi.org/10.4187/respcare.05366
4. Itamoto CH, Lima BT, Sato J, Fujita RR. Indications and complications of tracheostomy in children. *Braz J Otorhinolaryngol*. 2010;76(3):326-331. https://doi.org/10.1590/S1808-86942010000300010
5. Simon TD, Berry J, Feudtner C, et al. Children with complex chronic conditions in inpatient hospital settings in the United States. *Pediatrics*. 2010;126(4):647-655. https://doi.org/10.1542/peds.2009-3266
6. de Caen AR, Maconochie IK, Aickin R, et al.; Pediatric Basic Life Support and Pediatric Advanced Life Support Chapter Collaborators. Part 6: pediatric basic life support and pediatric advanced life support—2015 international consensus on cardiopulmonary resuscitation and emergency cardiovascular care science with treatment recommendations. *Circulation*. 2015;132(16)(suppl 1):S177-S203. https://doi.org/10.1161/CIR.0000000000000275
7. Bonvento B, Wallace S, Lynch J, Coe B, McGrath BA. Role of the multidisciplinary team in the care of the tracheostomy patient. *J Multidiscip Healthc*. 2017;10:391-398. https://doi.org/10.2147/JMDH.S118419
8. Morris LL, Whitmer A, McIntosh E. Tracheostomy care and complications in the intensive care unit. *Crit Care Nurse*. 2013;33(5):18-30. https://doi.org/10.4037/ccn2013518
9. Agarwal A, Marks N, Wessel V, et al. Improving knowledge, technical skills, and confidence among pediatric health care providers in the management of chronic tracheostomy using a simulation model. *Pediatr Pulmonol*. 2016;51(7):696-704. https://doi.org/10.1002/ppul.23355
10. Shah NH, Bhansali P, Barber A, et al. Children with medical complexity: a web-based multimedia curriculum assessing pediatric residents across North America. *Acad Pediatr*. 2018;18(1):79-85. https://doi.org/10.1016/j.acap.2017.08.008
11. Paul F. Tracheostomy care and management in general wards and community settings: literature review. *Nurs Crit Care*.

Copyright © 2020 Khan et al. This is an open-access publication distributed under the terms of the Creative Commons Attribution license.

2010;15(2):76-85. https://doi.org/10.1111/j.1478-5153.2010.00386.x

12. Everitt E. Tracheostomy 1: caring for patients with a tracheostomy. *Nurs Times*. 2016;112(19):16-20.

13. Yelverton JC, Nguyen JH, Wan W, Kenerson MC, Schuman TA. Effectiveness of a standardized education process for tracheostomy care. *Laryngoscope*. 2015;125(2):342-347. https://doi.org/10.1002/lary.24821

14. Passiment M, Sacks H, Huang G. *Medical Simulation in Medical Education: Results of an AAMC Survey*. Association of American Medical Colleges; 2011. Accessed September 20, 2019. https://store.aamc.org/medical-simulation-in-medical-education-results-of-an-aamc-survey-pdf.html

15. Thrasher J, Baker J, Ventre KM, et al. Hospital to home: a quality improvement initiative to implement high-fidelity simulation training for caregivers of children requiring long-term mechanical ventilation. *J Pediatr Nurs*. 2018;38:114-121. https://doi.org/10.1016/j.pedn.2017.08.028

16. Shah N, Bhansali P, McGarry M, et al. Simulation series: emergencies in technology-dependent children. *MedEdPORTAL*. 2013;9:9310. https://doi.org/10.15766/mep_2374-8265.9310

17. Tofil NM, Rutledge C, Zinkan JL, et al. Ventilator caregiver education through the use of high-fidelity pediatric simulators: a pilot study. *Clin Pediatr (Phila)*. 2013;52(11):1038-1043. https://doi.org/10.1177/0009922813505901

18. Downing SM, Yudkowsky R, eds. *Assessment in Health Professions Education*. Routledge; 2009.

19. Jaye P, Thomas L, Reedy G. “The Diamond”: a structure for simulation debrief. *Clin Teach*. 2015;12(3):171-175. https://doi.org/10.1111/tct.12300

20. Rudolph JW, Simon R, Dufresne RL, Raemer DB. There’s no such thing as “nonjudgmental” debriefing: a theory and method for debriefing with good judgment. *Simul Healthc*. 2006;1(1):49-55. https://doi.org/10.1097/01266021-200600110-00006

21. Zackoff MW, Real FJ, Abramson EL, Li STT, Klein MD, Gusic ME. Enhancing educational scholarship through conceptual frameworks: a challenge and roadmap for medical educators. *Acad Pediatr*. 2019;19(2):135-141. https://doi.org/10.1016/j.acap.2018.08.003

22. Khan EK, Meier M, Brinton J, Lockspeiser TM, Liptzin DR. Pulmonary Resident BootCamp: a novel subspecialty preparatory curriculum for pediatric residents. *Am J Respir Crit Care Med*. 2019;199:A1369. https://doi.org/10.1164/ajrccm-conference.2019.199.1_MeetingAbstracts.A1369

Received: October 16, 2019
Accepted: April 23, 2020
Published: October 1, 2020

Copyright © 2020 Khan et al. This is an open-access publication distributed under the terms of the Creative Commons Attribution license.
